# Scraps

**Published:** 1898-06

**Authors:** 


### SCRAPS.

PICKED UP BY THE ASSISTANT-EDITOR.

**Practical Dietetics.**—"My wife," he remarked, "has made a very important discovery." "Indeed! what is it?" "A new substance that is apparently indestructible." I recalled the fact that his wife had been a professor of natural sciences prior to her marriage, and inquired if she had been long at work upon the invention. "No," he replied, "it came about quite by accident. She was trying to make a sponge cake."—*Lippincott's Magazine*, quoted in *Medical Age*.

**Medical Philology** (XXVI).—The *Promptorium* has "Cruschylbone, or grystyl-bone. *Cartilago*." Crush is obsolete in English as a signification of cartilage or gristle, but Forby, in *The Vocabulary of East Anglia*, published in 1830-38, from which the *New English Dictionary* quotes, and to which Mr. Way's note in the *Promptorium* refers, gives "*Crish*, *Crush*, cartilage, or soft bones of young animals, easily crushed by the teeth," and "*Crush*, *crustle*, gristle." Mr. Way also says that "in Suffolk crussel or skrussel has a similar meaning."

The etymology of cartilage is unknown.

**"Sur un Médecin."**—The Latin epigrams, especially the medical ones, have been exceedingly difficult to equal for sting and conciseness. Here is one however, from the French of this century, attributed to François de Neufchâteau, which may take rank with them. It comes from page 249 of the 1892 issue of *La Lyre Française*, edited by Gustave Masson, and published by Macmillan and Co.:—

"Mes malades jamais ne se plaignent de moi,"
Disait un médecin d'ignorance profonde.
— Ah! repartit un plaisant, je le crois;
Vous les envoyez tous se plaindre en l'autre monde.

**Clinical Records** (23).—A description of the modern malady, Morbus Sabbatticus, or Sunday sickness, which we have somewhere met, is too good to lose. The peculiar features are: (1) That it only attacks members of the Church; (2) It never troubles them any day but Sunday; (3) The symptoms vary, but the patient can always sleep well the night before, and can always eat a hearty Sunday dinner shortly after an acute attack; (4) No case was ever known where the sick man was not able to be at his business early Monday; (5) It is generally the head of the family that is attacked; (6) When ladies are affected there will generally be found a complication with the dressmaker who failed to send the new garment on Saturday, or with a perfect fright of a bonnet; (7) In this disease no physician is summoned; (8) It never hurts the body, but it slays souls.—*Church Worker.*

**Medical "Bits of Old Bristol"** (7).—During the epidemics of the Black Death, the Jews were often accused of poisoning the wells from which the drinking water was obtained. It is therefore of special interest to note how the citizens of Bristol obtained their drinking water in the fourteenth century when the plague was rife.

The Rev. W. Hunt says that "the one redeeming feature in the sanitary condition of the town was that it had an abundant supply of water conveyed through conduits, for which it was largely indebted to its monasteries." (*Bristol*, Historic Towns Series, 1889, p. 78). A great deal of information on this subject will be found in a booklet on "The Pipes, Pumps, and Conduits of Bristol," consisting of articles written by Joseph Leech in *The Bristol Times and Felix Farley's Journal.* I give some notes about a portion of this water-supply, reserving for a future number the consideration of the remainder.

The house and grounds of the Carmelite Friars occupied the site extending from the Red Lodge in Park Row to the bottom of Colston Street and from Pipe Lane to the eastern end of Trenchard Street, where not long ago Steep Street existed. This foundation of the Carmelites dated from 1267, soon after which they obtained a supply of water from a spring on Brandon Hill. It was conveyed thence in a pipe which followed the course afterwards taken by Park Street and then passed along the line of Frogmore Street through Pipe Lane (to which it gave its name) to the Priory. From the cistern there a pipe afterwards supplied the parish of St. John, going through Host Street, over Frome Bridge, and then along Christmas Street into Broad Street, whence, about 1828, it was diverted to the spot in Nelson Street now familiar to all passers by.

**Obstetrics Home and Foreign** (5).—Henry, in his *History of Britain*, i. 459 tells us that "amongst the ancient Britons, when a birth was attended with any difficulty, they put certain girdles made for that purpose about the women in labour, which they imagined gave immediate and effectual relief. Such girdles were kept with care, till very lately, in many families in the Highlands of Scotland. They were impressed with several mystical figures, and the ceremony of binding them about the woman's waist was accompanied with words and gestures, which showed the custom to have been of great antiquity, and to have come originally from the Druids."—Brand's *Popular Antiquities*, ed. Bohn, 1849, vol. ii., p. 67.

The most reasonable of all the means adopted by primitive people to assist the patient in her labour is the steady compression of the abdomen and following down of the child in its descent. This is a feature common to the red, yellow, and black races, be it by compression of the fundus by the encircling arms of the husband, upon whose lap the patient rests; be it by the hands of one of the female assistants, sometimes from behind, sometimes from the front; or by a broad cloth or binder (California Indians and the natives of Southern India), which an assistant tightens during each pain—a treatment which has not yet lost the favour of obstetricians and was once quite popular. There are some who still place a towel about the abdomen of their patients, thinking to assist the descent of the child by the pressure exercised; it seems both to correct the direction of the child's descent and to hasten its passage. In its extreme and worst feature, we see this method of treatment exemplified by the Siamese, who seek to force the expulsion of the fœtus in difficult cases by permitting the attendant to trample upon the abdomen of the patient, who is lying prone upon her back. All primitive people resort to expression in one way or another. The Finns, in tedious cases, compress the abdomen by a belt or binder of some kind, or by holding the patient up, suspended, and shaking her as they would a pillow out of its case—a proceeding which is more efficient than mild, and serves as a last resort to the natives of Mexico, as well as other far distant people.—Engelmann's *Labor among Primitive Peoples*, 1882, p. 132.

